# Subcortical auditory system in tinnitus with normal hearing: insights from electrophysiological perspective

**DOI:** 10.1007/s00405-024-08583-3

**Published:** 2024-03-30

**Authors:** Hasan Colak, Eser Sendesen, Meral Didem Turkyilmaz

**Affiliations:** 1 Biosciences Institute, Newcastle University, Newcastle Upon Tyne, UK; 2 Department of Audiology, Hacettepe University, Ankara, Turkey

**Keywords:** Tinnitus, Frequency following response, Speech ABR, Speech-in-noise, Cochlear synaptopathy, Subcortical auditory system

## Abstract

**Purpose:**

The mechanism of tinnitus remains poorly understood; however, studies have underscored the significance of the subcortical auditory system in tinnitus perception. In this study, our aim was to investigate the subcortical auditory system using electrophysiological measurements in individuals with tinnitus and normal hearing. Additionally, we aimed to assess speech-in-noise (SiN) perception to determine whether individuals with tinnitus exhibit SiN deficits despite having normal-hearing thresholds.

**Methods:**

A total 42 normal-hearing participants, including 22 individuals with chronic subjective tinnitus and 20 normal individuals, participated in the study. We recorded auditory brainstem response (ABR) and speech-evoked frequency following response (sFFR) from the participants. SiN perception was also assessed using the Matrix test.

**Results:**

Our results revealed a significant prolongation of the O peak, which encodes sound offset in sFFR, for the tinnitus group (*p* < 0.01). The greater non-stimulus-evoked activity was also found in individuals with tinnitus (*p* < 0.01). In ABR, the tinnitus group showed reduced wave I amplitude and prolonged absolute wave I, III, and V latencies (*p* ≤ 0.02). Our findings suggested that individuals with tinnitus had poorer SiN perception compared to normal participants (*p* < 0.05).

**Conclusion:**

The deficit in encoding sound offset may indicate an impaired inhibitory mechanism in tinnitus. The greater non-stimulus-evoked activity observed in the tinnitus group suggests increased neural noise at the subcortical level. Additionally, individuals with tinnitus may experience speech-in-noise deficits despite having a normal audiogram. Taken together, these findings suggest that the lack of inhibition and increased neural noise may be associated with tinnitus perception.

**Supplementary Information:**

The online version contains supplementary material available at 10.1007/s00405-024-08583-3.

## Introduction

Tinnitus is defined as a phantom sound perception without any external stimulus. Up to 5% of the general population suffers from chronic tinnitus [1]. The principal risk factor for tinnitus is hearing loss, with approximately 90% of individuals with tinnitus having elevated audiometric thresholds [2]. This shows that hearing loss is an important factor for tinnitus perception; however, tinnitus can also occur in individuals with a normal audiogram. Recent work has proposed cochlear synaptopathy and extended high-frequency (> 8 kHz) hearing loss as potential explanations for normal audiogram findings in tinnitus [3, 4].

Although the exact mechanism remains unknown, subclinical peripheral deafferentations and the subsequent lack of input may trigger alterations throughout the central auditory pathway. This deafferentation leads to reduced activity within the cochlear nerve, subsequently causing down-regulation of inhibitory processes [5]. Studies have associated impaired inhibitory mechanisms with decreased GABAergic inhibition [6]. This deficit in inhibition may result in an increase in the spontaneous firing rate (SFR) within the auditory pathway, contributing to tinnitus perception. Animal studies have reported increased SFR and hyperactivity in the cochlear nucleus, inferior colliculus, and auditory thalamus [7, 8].

Previous work on tinnitus mechanisms has found increased SFR, central gain, neural synchrony, and prediction errors at the subcortical level, emphasizing its importance in tinnitus perception [9–13]. These findings suggest that individuals with tinnitus may show abnormalities within the subcortical auditory system. In this study, we collected speech-evoked frequency following response (sFFR) and ABR to evaluate subcortical auditory system in individuals with tinnitus and normal hearing. The sFFR mainly originates from subcortical auditory areas [14] and is sensitive to plastic changes in the auditory system [15]. The two main components of sFFRs are the temporal domain and the spectral domain. The temporal domain represents time-dependent changes in sFFR and typically consists of 7 peaks (V, A, C, D, E, F, and O) in response to 40 ms/da/stimuli. The spectral domain represents frequency encoding, providing information about F0 and its higher frequency harmonic components. Experience-led differences, including music training, speaking a tonal language, and bilingualism have been observed in sFFR [16–18]. However, the impact of negative experiences, such as tinnitus, on sFFR remains unclear. Omidvar et al. [19] reported prolonged sFFR peaks in tinnitus, whereas no difference was observed in ABR. Recently, Krizman et al. [20] showed that the non-stimulus-evoked activity component of the sFFR is driven by neural noise. It is plausible to anticipate increased neural noise due to increased spontaneous neural activity in tinnitus. Therefore, we also investigated the non-stimulus-evoked activity in the sFFR of individuals with tinnitus.

The subcortical auditory system, particularly the inferior colliculus and auditory thalamus, plays an important role in tinnitus perception, and sFFR is a reliable predictor of its functioning. However, sFFR is mostly overlooked in tinnitus work, and studies have focused only on ABR, measuring the lower part of the subcortical auditory system. Here, we aim to provide a comprehensive evaluation of the subcortical auditory system in normal-hearing individuals with tinnitus, while also considering their EHF hearing. We hypothesize that individuals with tinnitus show impaired functioning of the subcortical auditory system and increased neural noise compared to control participants. Speech-in-noise (SiN) perception is an important daily life ability that can affect quality of life. SiN difficulty is commonly reported among individuals with tinnitus [21]. While some studies have found SiN deficits among individuals with tinnitus despite having normal hearing [22, 23], others have reported no such deficits [24, 25]. Given this discrepancy, we also aim to investigate SiN performance using a sentence recognition test in individuals with tinnitus and normal hearing.

## Materials and methods

### Participants

Twenty-two individuals (14 female and 8 male) with normal-hearing chronic tinnitus (more than 6 months) and 20 normal individuals (13 female and 7 male) participated in the study. The mean age of individuals with chronic tinnitus was 31.0 years (SD 7.3 years, range 19–49), while the mean age of normal individuals was 29.6 years (SD 8.6 years, range 19–47). Seventeen tinnitus patients reported bilateral tinnitus, while five patients experienced tinnitus in the right ear. Individuals with any organic or neurological cause of tinnitus were excluded from the study. Hornickel et al. [26] found earlier latencies and more robust spectral encoding in the right ear sFFR compared to left ear sFFR recordings. Therefore, all tests were conducted on the right ear to eliminate differences between ears in electrophysiological testing. Additionally, we excluded individuals who experienced tinnitus only in their left ear to control for the right ear advantage. All participants underwent audiometric evaluation ranging from 0.25 kHz to 16 kHz using TDH 39 (0.25–8 kHz) and Sennheiser HDA 200 circumaural headphones (10, 12.5, 14, and 16 kHz). Individuals with normal hearing (thresholds ≤ 20 dB HL) in the standard pure-tone audiometric evaluation (0.25–8 kHz) were included in the study and, all participants had normal middle ear functions (type A tympanogram). There was no asymmetry in audiometric evaluation between the ears (≤ 10 dB). To ensure the absence of mild cognitive impairment, all participants completed the Montreal Cognitive Assessment (MoCA) and were found to be within normal limits. Individuals with tinnitus also completed the Tinnitus Handicap Inventory developed by Newman et al. [27] to assess the severity of tinnitus in daily life.

### ABR recordings

ABR recordings were collected using the Intelligent Hearing System (IHS) device. Stimulus presentation was conducted using ER-3A (Etymotic Research) insert earphones. Non-inverting, inverting and, ground electrodes were located at Cz, A2, and Fz, respectively. Electrode impedances were kept below 3 kOhm. ABRs were recorded from the right ear using 100 ms click stimulus with alternating polarity. We presented a stimulus intensity of 90 dB nHL (~ 125peSPL) to evaluate low spontaneous rate fibers, which encode high-intensity levels. The stimulus presentation rate was set at 21.1 Hz, and two blocks of 3000 artifact-free sweeps were collected from each participant. Recordings were filtered using a 30–1500 Hz band-pass filter. Subsequently, the ABR recordings were analyzed using the IHS device. Two expert audiologists, blinded to the participant's tinnitus status, served as peak selectors for determining waves I, III, and V.

### SFFR recordings

The participants’ sFFR data were collected using the IHS complex ABR module, with the same electrode placement and earphone configuration as for ABR recording. sFFR traces were recorded in the same session as ABR. To minimize interference from high electrode impedance with non-stimulus-evoked activity and ensure high-quality recordings, we employed more conservative electrode impedance criteria (< 3 kOhm). A 40 ms/da/stimulus was presented to the right ear of participants at a rate of 10.9 Hz and an intensity level of 80 dB SPL, with alternating polarity. Artifact rejection criteria were set at ± 31 µV, and recordings were online filtered with a 100–2000 Hz band-pass filter. Two blocks of 3000 artifact-free sweeps were collected and added. ABR and sFFR data were recorded while participants were seated in a relaxed chair in a Faraday cage room.

ABR and sFFR recordings were analyzed using the IHS device and MATLAB. Two expert audiologists were peak pickers in the determination of peaks. The temporal domain of the sFFR consists of seven peaks (Vn-A, C, D, E, F, and O). We excluded peak C from the analysis, because it may not be detectable even in normal individuals [28]. Further analysis and peak-to-peak amplitude calculations of the sFFR data were conducted using the Brainstem Toolbox [28] in MATLAB. Pre-stimulus RMS (root-mean-square) values were calculated as a marker of non-stimulus-evoked activity. Additionally, the fast Fourier transform was employed to spectrally analyze the data in three frequency ranges: 103–125 Hz for the fundamental frequency (F0), 454–720 Hz for the first formant (F1), and 721–1200 Hz for the higher frequency harmonics.

### SiN assessment

SiN performance was assessed using Matrix test [29], which is a sentence recognition test consisting of five-word sentence structures. Randomly selected sentences were presented to participants in speech-shaped noise. Matrix test thresholds were calculated using an adaptive procedure in otosuite software based on a mathematical formula [30]. Lower dB signal-to-noise ratio (SNR) scores indicate better performance, while higher dB SNR scores indicate poorer performance. Stimulus presentation was conducted using Sennheiser HDA 200 circumaural headphones. SiN perception was assessed under two conditions: right speech right noise (RsRn) and binaural speech binaural noise (binaural). Two training lists were used to familiarize participants with the task.

### Statistical analyses

In this study, G*Power software was used to determine the sample size of participants. Statistical analyses were performed using the SPSS version 26. The Shapiro–Wilk test was used to evaluate the normality of the data, and Levene’s test was used to assess the homogeneity of variances. To compare two groups, an independent samples *t* test was conducted. Cohen’s *d* was also calculated to show effect size. Multivariate normality was assessed using the Henze–Zirkler test. Multivariate analysis of variance (MANOVA) and Roy’s largest root test were performed for the two SiN listening conditions. Correlations between THI scores, electrophysiological tests, and SiN perception were evaluated using Pearson’s correlation coefficient. The statistical significance level was set at *p* < 0.05 for all data.

## Results

### Audiometric findings

**Fig. 1:**
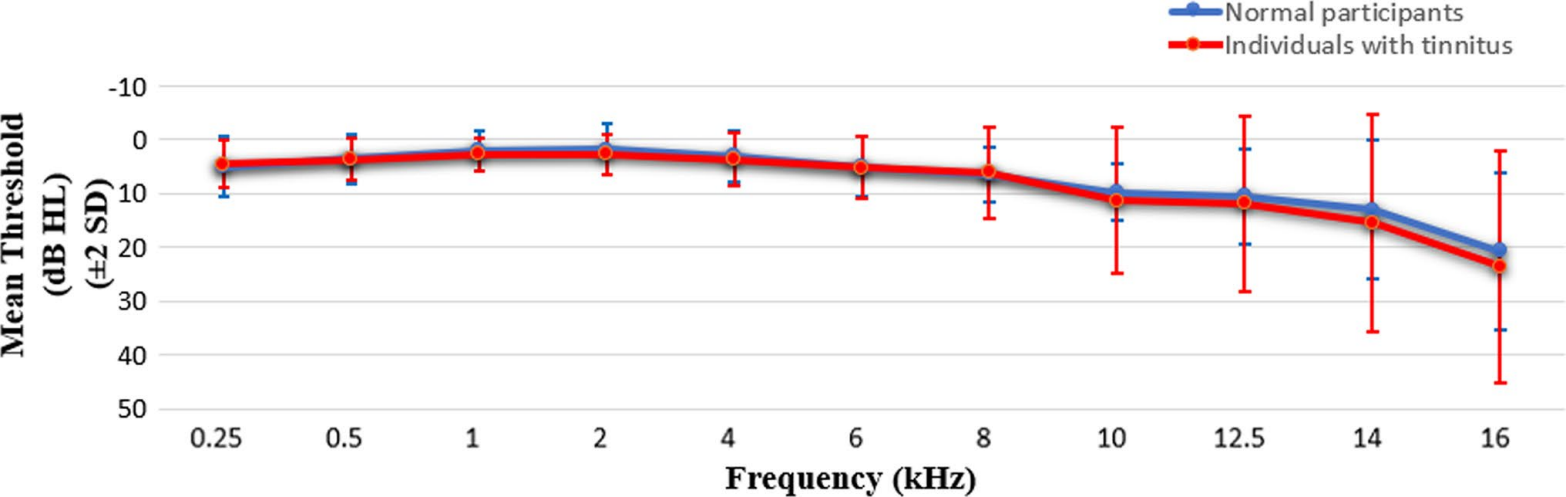
Comparison of mean audiometric thresholds between the individuals with tinnitus and normal participants

Twenty-two individuals with tinnitus and 20 normal participants included to present study. Comparison of the two groups showed that age did not differ significantly between the groups (*p* = 0.589). Other demographic features and tinnitus characteristic of the participants are presented in Table 1. Audiometric thresholds were obtained between 0.25 and 16 kHz frequencies. Our participants exhibited normal hearing (≤ 20 dB) in standard audiometric frequencies ranging from 0.25 to 8 kHz. Comparison of the two groups revealed no significant differences in this frequency range (*p* ≥ 0.454). In the extended high-frequency (EHF) range (8–16 kHz), the tinnitus group showed slightly higher hearing thresholds compared to the control group; however, this difference was not statistically significant for any frequency within the EHF range (*p* ≥ 0.478). Audiometric thresholds of the participants are shown in Fig. 1.

**Table 1:** Demographic features and tinnitus characteristics of the participants

	Individuals with tinnitus (*n* = 22)	Normal participants (*n* = 20)
Age (range)	31.0 $\pm 7.35$ (19–49)	29.6 $\pm 8.6$ (19–47)
Gender		
Male	8 (37%)	7 (35%)
Female	14 (63%)	13 (65%)
Tinnitus Lateralization		
Right ear	5 (23%)	
Bilateral	17 (77%)	
Tinnitus duration (year)	3.27 $\pm 2$.63	
Tinnitus Handicap Inventory	58.5 ± 21.6	
Tinnitus frequency (Hz)	4704 $\pm$ 2954	
Tinnitus intensity (HL)	34.3 $\pm$ 6.4	
Minimum masking level	51.8 $\pm$ 10.2	
Residual inhibition		
No	15 (68%)	
Yes	7 (32%)	

*N* number of participants

### Electrophysiological findings

**Fig. 2:**
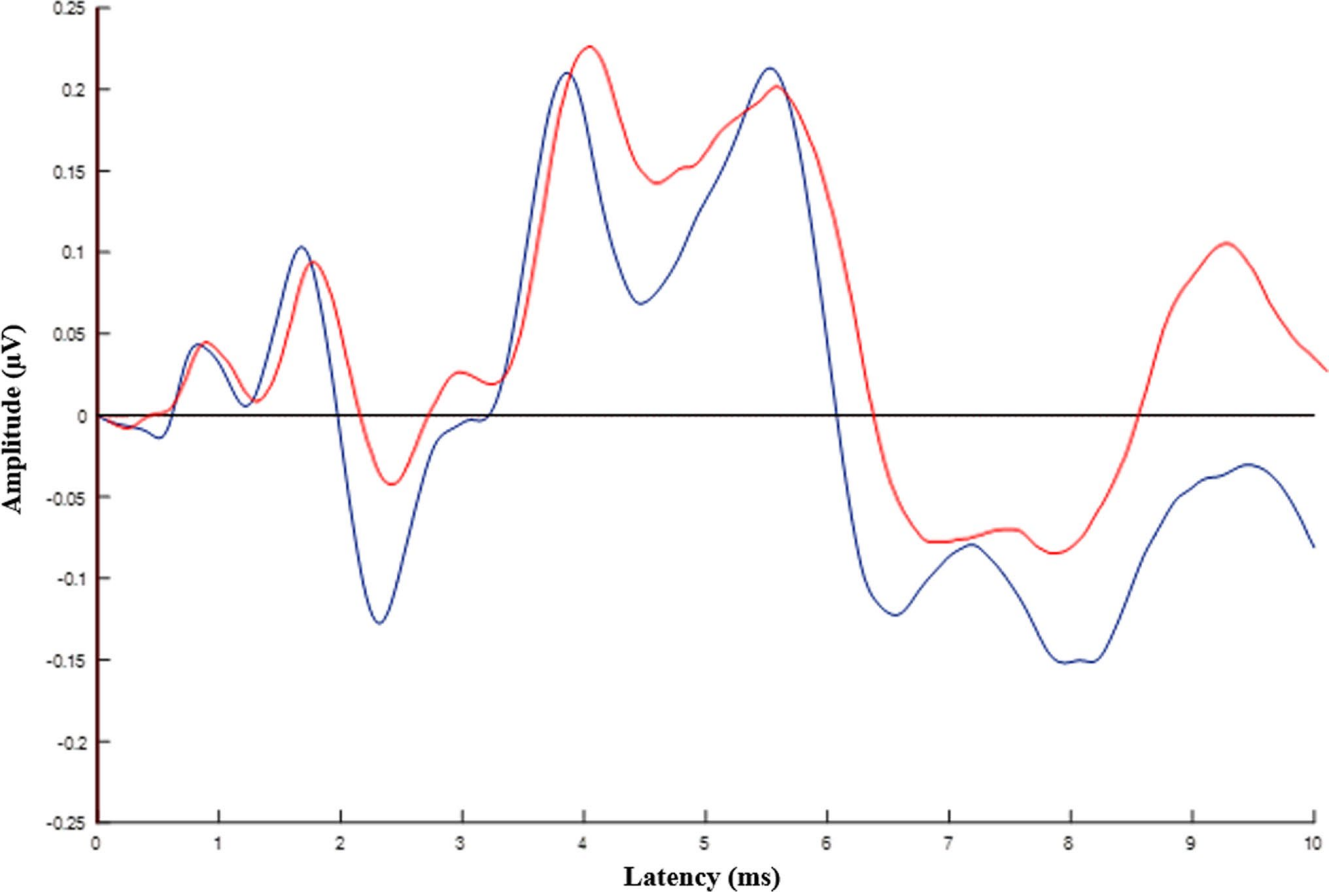
Grand averaged ABR waveforms of the individuals with tinnitus (red) and normal participants (blue)

**Fig. 3:**
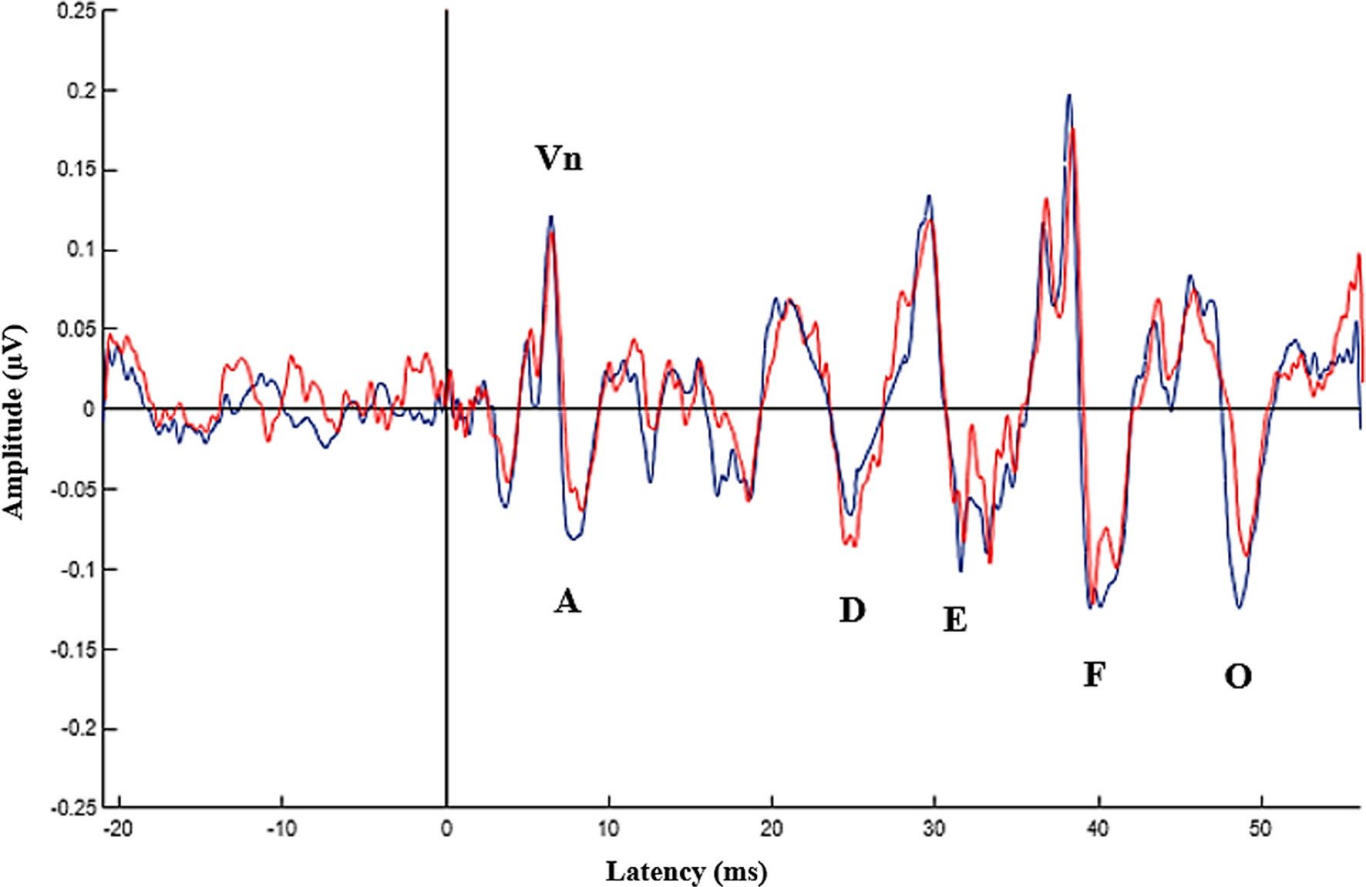
Grand averaged sFFR waveforms of the individuals with tinnitus (red) and normal participants (blue)

Table 2 displays the ABR findings of the participants. The ABR findings show that absolute latencies of wave I, III, and V were longer in individuals with tinnitus (*p* ≤ 0.02). In addition, wave I amplitude was significantly reduced in individuals with tinnitus (*p* = 0.004). Figure 2 shows the grand averaged ABR waveforms of the participants. We also collected sFFR data from the participants, with the temporal and spectral domain aspects presented in Table 3. Individuals with tinnitus showed significantly longer latencies in the O peak of sFFR, which encodes sound offset (*p* = 0.009). However, there was no significant difference in spectral encoding between the tinnitus and control groups (*p* ≥ 0.16). Further, the pre-stimulus RMS value, an indicator of non-stimulus-evoked activity, was significantly higher in the tinnitus group (*p* = 0.001). Figure 3 illustrates the grand averaged sFFR waveforms of the participants. Pearson correlation analysis revealed that THI scores were not significantly correlated with any ABR or sFFR components (*p* > 0.05).

**Table 2:** ABR findings of the individuals with tinnitus and normal participants

	Participants			
	Individuals with tinnitus (*n* = 22)	Normal participants (*n* = 20)		
	Mean $\pm$ SD	Mean $\pm$ SD	Effect size (Cohen’s *d*s)	*p*
ABR latencies (ms)				
I	1.88 $\pm$ 0.21	1.73 $\pm$ 0.07	0.93	0.01*
III	4.01 $\pm$ 0.17	3.85 $\pm$ 0.13	1.05	0.004**
V	5.80 $\pm$ 0.24	5.65 $\pm$ 0.16	0.71	0.02*
I–III	2.12 $\pm$ 0.14	2.11 $\pm$ 0.16	0.06	0.74
III–V	1.80 $\pm$ 0.16	1.77 $\pm$ 0.11	0.21	0.43
I–V	3.93 $\pm$ 0.23	3.83 $\pm$ 0.30	0.37	0.24
ABR amplitudes (µV)				
I	0.15 $\pm$ 0.08	0.25 $\pm$ 0.11	− 1.04	0.004**
III	0.16 $\pm$ 0.06	0.18 $\pm$ 0.08	− 0.28	0.37
V	0.34 $\pm$ 0.15	0.40 $\pm$ 0.14	− 0.41	0.27
V/I	2.51 $\pm$ 1.08	1.96 $\pm$ 1.30	0.46	0.15

*N* number of participants, *SD* standard deviation, *p < .05, **p < .01

**Table 3:** sFFR findings of the individuals with tinnitus and normal participants

	Participants			
	Individuals with tinnitus (*n* = 22)	Normal participants (*n* = 20)		
	Mean $\pm$ SD	Mean $\pm$ SD	Effect size (Cohen’s *d*s)	*p*
sFFR latencies (ms)				
Vn	6.66 $\pm$ 0.34	6.51 $\pm$ 0.28	0.47	0.09
A	7.61 $\pm$ 0.43	7.50 $\pm$ 0.33	0.28	0.47
D	22.55 $\pm$ 0.62	22.45 $\pm$ 0.56	0.16	0.57
E	31.01 $\pm$ 0.41	30.82 $\pm$ 0.48	0.42	0.19
F	39.51 $\pm$ 0.44	39.35 $\pm$ 0.37	0.39	0.23
O	48.57 $\pm$ 0.45	48.20 $\pm$ 0.39	0.87	0.009**
sFFR amplitudes (µV)				
Vn/A	0.27 $\pm$ 0.08	0.28 $\pm$ 0.09	− 0.11	0.90
D	0.17 $\pm$ 0.08	0.17 $\pm$ 0.07	0	0.74
E	0.25 $\pm$ 0.12	0.25 $\pm$ 0.08	0	0.91
F	0.34 $\pm$ 0.13	0.36 $\pm$ 0.10	− 0.15	0.60
O	0.21 $\pm$ 0.07	0.24 $\pm$ 0.11	− 0.32	0.19
sFFR spectral amplitudes (µV)				
F0	0.035 $\pm$ 0.015	0.034 $\pm$ 0.013	0.07	0.81
F1	0.010 $\pm$ 0.003	0.011 $\pm$ 0.02	− 0.07	0.16
Higher frequency harmonics	0.003 $\pm$ 0.001	0.003 $\pm$ 0.001	0	0.75
Pre-stimulus RMS^a^	0.075 $\pm$ 0.02	0.057 $\pm$ 0.009	1.14	0.001**

*N* number of participants, *SD* standard deviation, *RMS* root mean square, *p < .05, **p < .01

^a^Non-stimulus-evoked activity

### SiN findings

**Fig. 4:**
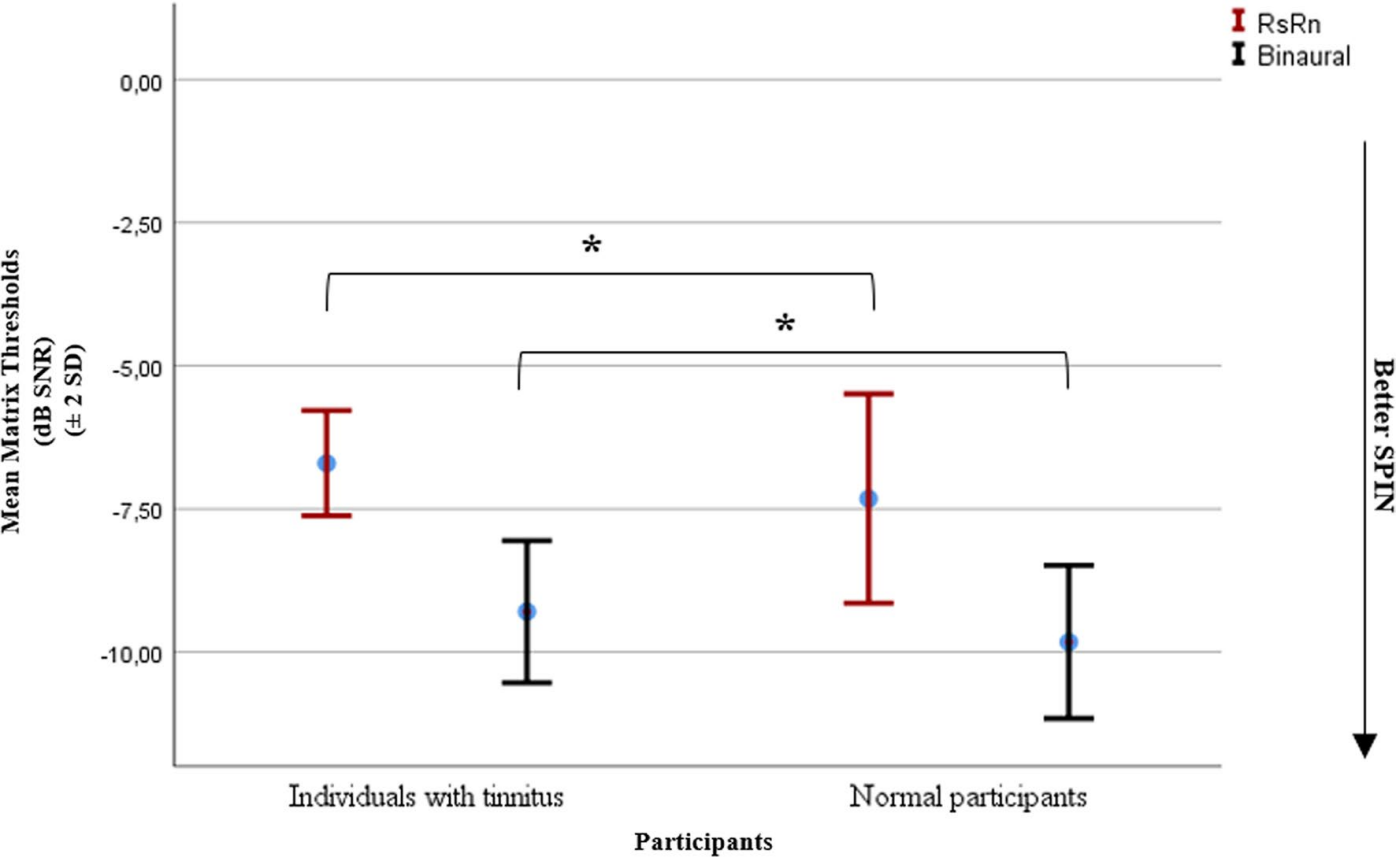
Speech-in-noise (Matrix) thresholds (± 2 SD) of individuals with tinnitus and normal participants

SiN perception of the participants was evaluated in two listening conditions, RsRn and binaural. MANOVA analysis showed a significant poorer performance in individuals with tinnitus compared to normal participants (*p* = 0.014, see Fig. 4). In RsRn condition, the mean matrix threshold was − 6.70 ± 0.45 dB SNR for individuals with tinnitus, whereas it was − 7.32 ± 0.91 dB SNR for normal participants (Cohen’s *d* = 0.85, *p* = 0.011). The mean threshold was − 9.29 ± 0.62 dB SNR for individuals with tinnitus and − 9.82 ± 0.66 dB SNR for normal participants in binaural condition (Cohen’s *d* = 0.82, *p* = 0.011). There was no correlation between SiN perception and THI score (*p* > 0.05).

## Discussion

In this study, our aim was to evaluate the subcortical auditory system and assess speech-in-noise (SiN) performance in individuals with tinnitus and normal hearing. Our results indicated that individuals with tinnitus exhibited a decreased amplitude in wave I and prolonged latencies for wave I, III, and V in ABR. We also found delayed O peaks and increased non-stimulus-activity, likely suggesting impaired inhibitory mechanisms in tinnitus. Despite having normal hearing within standard audiometric frequencies (0.25–8 kHz), the tinnitus group showed poorer SiN perception.

There is a growing body of literature which has identified that lifelong experiences modulate sFFR [15, 17, 31]. Here, we are interested in showing tinnitus-related differences in the upper part of the subcortical auditory system using sFFR. According to our findings, amplitude of the peaks did not differ significantly; however, individuals with tinnitus had significant delay in peak O, which indicates the encoding of sound offset. Previous work on the offset response suggests that there may be a particular sound offset pathway in the auditory system that reaches from brainstem to auditory cortex including inferior colliculus and auditory thalamus that can be important for tinnitus generation [32–34]. Kopp-Scheinpflug et al. [33] also proposed that the offset response may be particularly sensitive to alterations in the excitation–inhibition balance. Animal studies have indicated that projections between the sound offset pathways are connected by GABAergic inhibitory neurons [35, 36]. It is plausible to argue that a well-functioning inhibitory mechanism is required for the accurate encoding of the sound offset. So far, previous animal studies have shown that inhibitory neurotransmission is reduced in tinnitus due to decreased GABAergic inhibition at the subcortical level [37, 38]. It is likely that hyperactivity associated with reduced inhibition may lead to tinnitus and result in abnormal encoding of sound offset responses.

A recent study by Krizman et al. [20] showed that non-stimulus-activity in sFFR mostly arises from background neural activity rather than non-neural noise. In this study, we found greater non-stimulus-activity in individuals with tinnitus, which may be associated with increased neural noise in tinnitus. Increased spontaneous activity in tinnitus has already demonstrated by animal studies [39, 40]. Consistent with these findings, our results show that individuals with tinnitus, despite having normal audiometric thresholds, exhibit increased background neural activity at the subcortical level. This finding may also suggest an impaired inhibition mechanism at the subcortical level as proposed here. Further, it is also possible to speculate that tinnitus perception may rely on increased neural noise, in which weakened inhibitory mechanism serves as a predisposition to tinnitus.

Elevated hearing thresholds or cochlear synaptopathy can lead to deafferentation and a lack of input to the subcortical auditory system. According to the concept of stochastic resonance, neurons tend to maintain their firing rates, resulting in increased neural noise [41]. It should be noted that increased noise is associated with increased SFR and can emerge even in the absence of stimuli. Conversely, increased central gain is related to stimulus-evoked activity and it requires a presence of a stimulus. We therefore might expect to find increased amplitudes in ABR and sFFR in the tinnitus group. The possible reason for this finding may lie in the fact that none of our participants reported hyperacusis, suggesting that increased central gain in tinnitus might be associated more with hyperacusis rather than solely with tinnitus at the subcortical level [2, 42]. Recently, Zeng [43] developed a mathematical modeling and proposed a central variance mechanism that suggests the contribution of increased neural noise, rather than gain, in tinnitus. Our study may provide evidence supporting Zeng’s tinnitus modeling, which suggests increased neural noise in individuals with tinnitus.

Previous studies have showed abnormalities in ABR findings in individuals with tinnitus and normal hearing [44, 45]. Our findings also indicate prolonged absolute latencies of waves I, III, and V, along with decreased wave I amplitude in individuals with tinnitus. It is likely that the delay in wave I latency and the decrease in amplitude may result from cochlear synaptopathy or other forms of peripheral deafferentation. There is ongoing discussion about the cochlear synaptopathy and its clinical significance. Guest et al. [46] suggested that EHF may interfere with ABR wave I generation, potentially leading to a reduction in wave I amplitudes. However, in this study, the fact that individuals with tinnitus and control participants had similar EHF thresholds suggests that any possible influence related to EHF hearing loss on our results is less likely. It is important to bear in mind that our results cannot rule out the influence of other factors such as large inter-individual variation and the limited reliability of scalp recorded brainstem responses, which are reported limitations of ABR recordings in cochlear synaptopathy studies, particularly in wave I interpretation [47]. Although prolonged latencies in waves I, III, and V are commonly observed in conductive hearing loss [48], our participants showed normal tympanometry findings. It is possible that a prolongation in wave I may cause a delay in the absolute latencies of subsequent waves without affecting the interpeak latencies.

In the present study, consistent with Gilles et al. [22], we found poorer SiN perception in individuals with tinnitus. Recent work on EHF showed an association between EHF hearing loss and SiN deficit. [49, 50]. The fact that both groups in our study had similar EHF thresholds raises the possibility that SiN deficits may be related to subclinical factors, such as cochlear synaptopathy or auditory cognitive dysfunction due to tinnitus. Several cognitive predictors, such as working memory, attention, and auditory grouping, have been linked to SiN perception [51]. Rossiter et al. [52] showed that individuals with tinnitus may experience working memory and attention deficits. Therefore, although we did not assess these cognitive factors in our study, they may potentially contribute to the poorer SiN performance observed in individuals with tinnitus.

## Limitations and future directions

As previously mentioned, our study did not investigate the cognitive predictors of SiN perception. Tinnitus-related deficits in cognitive processes may be the primary cause of SiN deficit in individuals with tinnitus. Further studies are necessary to elucidate the main underlying factors contributing to SiN deficits in tinnitus. Large inter-individual variability in scalp recorded ABR wave I may be misleading to provide direct evidence to cochlear synaptopathy. Therefore, using electrocochleography, especially promontory recorded, may offer more reliable results to indicate cochlear synaptopathy in future studies. The sensitivity of sFFR to differences in the subcortical auditory system adds significant value, potentially making it a valuable tool in the management of tinnitus in clinical practice. Further studies focusing on its clinical role in tinnitus management are needed.

## Conclusion

In conclusion, our results may indicate impaired subcortical auditory processing in individuals with tinnitus, despite their normal hearing. We observed a significant prolongation of the O peak in the tinnitus group, suggesting a deficit in sound offset encoding possibly due to impaired inhibitory mechanisms. We also found greater non-stimulus-evoked activity in individual with tinnitus, indicating increased neural noise. Moreover, the tinnitus group exhibited reduced wave I amplitude and prolonged latencies for waves I, III, and V in ABR, despite having normal tympanogram results. This may show that deafferentation within the early stages of the auditory pathway may be linked to tinnitus and auditory temporal abnormalities in the upper part of the subcortical auditory system. Taken together, our findings provide evidence suggesting that lack of inhibition and increased neural noise may play a role in tinnitus perception. Further, it appears that individuals with tinnitus may experience speech-in-noise deficits despite having normal-hearing thresholds.

### Supplementary Information

Below is the link to the electronic supplementary material.

Supplementary file1 (DOCX 18 KB)

## Data Availability

The data that support the findings of this study are available from the corresponding author, (H. C.), upon request.
