# Supplementary material for: Subcortical auditory system in tinnitus with normal hearing: insights from electrophysiological perspective
Source: Eur Arch Otorhinolaryngol. 2024 Mar 30;281(8):4133–42. doi: 10.1007/s00405-024-08583-3 (PMC11266230; doi:10.1007/s00405-024-08583-3)
Supplement: Supplementary file 1 — Supplementary file1 (DOCX 18 KB) [file 405_2024_8583_MOESM1_ESM.docx]

**Table.** Audiometric thresholds of the individuals with tinnitus and control group.

| **Frequency (kHz)** | **Audiometric Thresholds (dB HL)**  **Mean**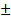**SD** | | ***p*** |
| --- | --- | --- | --- |
|  | **Control Participants**  **(n = 20)** | **Individuals with Tinnitus**  **(n = 22)** |  |
| **0.25** | 5.0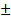5.61 | 4.54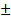4.33 | *0.77* |
| **0.5** | 3.5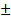3.61 | 3.63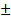3.83 | *0.91* |
| **1** | 2.0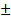3.76 | 2.72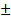2.97 | *0.49* |
| **2** | 1.75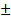4.66 | 2.72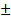3.69 | *0.45* |
| **4** | 3.0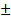4.7 | 3.63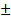4.92 | *0.67* |
| **6** | 5.0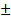5.61 | 5.22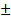6.98 | *0.90* |
| **8** | 6.5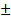5.15 | 6.13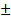8.44 | *0.86* |
| **10** | 9.75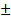5.25 | 11.36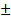14.15 | *0.63* |
| **12.5** | 10.5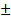8.87 | 11.81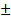16.22 | *0.74* |
| **14** | 13.0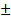13.01 | 15.45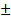20.11 | *0.64* |
| **16** | 20.75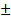14.62 | 23.63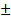21.44 | *0.61* |
| **PTA** | 2.56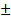2.76 | 3.18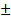2.82 | *0.47* |
| **EHFPTA** | 13.53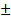8.33 | 15.56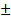16.73 | *0.62* |

SD: Standard deviation, PTA (pure tone average for 0.5, 1, 2, and 4 kHz), EHFPTA (extended high frequency pure tone average for 10, 12.5, 14, and 16 kHz)
